# An Adaptive Defect Weighted Sampling Algorithm to Design Pseudoknotted RNA Secondary Structures

**DOI:** 10.3389/fgene.2016.00129

**Published:** 2016-07-22

**Authors:** Kasra Zandi, Gregory Butler, Nawwaf Kharma

**Affiliations:** ^1^Computer Science Department, Concordia UniversityMontreal, QC, Canada; ^2^Centre for Structural and Functional Genomics, Concordia UniversityMontreal, QC, Canada; ^3^Electrical and Computer Engineering Department, Concordia UniversityMontreal, QC, Canada

**Keywords:** RNA secondary structure, sequence design algorithm, pseudoknot, hammerhead ribozyme, Pseudobase

## Abstract

Computational design of RNA sequences that fold into targeted secondary structures has many applications in biomedicine, nanotechnology and synthetic biology. An RNA molecule is made of different types of secondary structure elements and an important RNA element named pseudoknot plays a key role in stabilizing the functional form of the molecule. However, due to the computational complexities associated with characterizing pseudoknotted RNA structures, most of the existing RNA sequence designer algorithms generally ignore this important structural element and therefore limit their applications. In this paper we present a new algorithm to design RNA sequences for pseudoknotted secondary structures. We use NUPACK as the folding algorithm to compute the equilibrium characteristics of the pseudoknotted RNAs, and describe a new adaptive defect weighted sampling algorithm named Enzymer to design low ensemble defect RNA sequences for targeted secondary structures including pseudoknots. We used a biological data set of 201 pseudoknotted structures from the Pseudobase library to benchmark the performance of our algorithm. We compared the quality characteristics of the RNA sequences we designed by Enzymer with the results obtained from the state of the art MODENA and antaRNA. Our results show our method succeeds more frequently than MODENA and antaRNA do, and generates sequences that have lower ensemble defect, lower probability defect and higher thermostability. Finally by using Enzymer and by constraining the design to a naturally occurring and highly conserved Hammerhead motif, we designed 8 sequences for a pseudoknotted *cis*-acting Hammerhead ribozyme. Enzymer is available for download at https://bitbucket.org/casraz/enzymer.

## 1. Introduction

Ribonucleic acid (RNA) molecules play critical roles in various key cellular processes. Other than the messenger RNA (mRNA) (Singer and Leder, 1966) several other classes of RNAs have been discovered to be functional and the pace of discovery has accelerated over the past decade (Stark et al., 2007; Stefani and Slack, 2008; Fu et al., 2013; Roth et al., 2014). Functional RNAs are termed non-coding RNAs (ncRNAs) because they perform their functionality directly and not via their protein products (Mattick and Makunin, 2006). NcRNAs are involved in translation (tRNA) (Giegé et al., 1993), splicing (snRNA) (Matera and Wang, 2014), processing of other RNAs (snoRNA) (Bratkovič and Rogelj, 2014) and other key regulatory processes (Hannon, 2002; Bartel, 2009; Smith et al., 2010; Scarborough et al., 2014).

Due to their diverse range of functionalities, ncRNA are well suited for applications in synthetic biology (Khalil and Collins, 2010; Liang et al., 2011; Rodrigo et al., 2013), therapeutics (Lainé et al., 2011; Burnett and Rossi, 2012; Shum and Rossi, 2013), as well as nanotechnology (Afonin et al., 2013; Geary et al., 2014). The functional form of ncRNAs often requires a specific 3D structure (Shapiro et al., 2007) that is primarily determined by the secondary structure, as well as the sequence composition of the molecule (Leontis and Westhof, 2003; Dieterich and Stadler, 2013). Despite the difficulties of determining the 3D structure of RNAs, secondary structure prediction and secondary structure classification provide a major key in determining the potential functions (Laing and Schlick, 2011) as well as family signature (Griffiths-Jones et al., 2005) of the ncRNA molecules. Hence, developing better methods to design RNA sequences with specified secondary structures is a valuable pursuit as it opens doors to multiple applications.

The problem of designing artificial RNA sequences that fold into a targeted secondary structure is computationally difficult (Schnall-Levin et al., 2008; Haleš et al., 2015) and most of the existing methods resort to heuristics and stochastic local search strategies. The widely used RNA design strategy consists of two steps: first a random seed is generated; next, this seed is iteratively mutated until it adopts the desired folding properties as predicted by a folding algorithm such as RNAfold (Hofacker, 2003), mfold (Zuker, 2003) or CentroidFold (Hamada et al., 2009).

RNAinverse (Hofacker, 2003) is one of the first and most widely used RNA secondary structure design programs. RNAinverse decomposes the given target structure into smaller subunits and attempts to find an RNA sequence by an adaptive local walk, or greedy algorithm. The initial seed sequence is randomly chosen; then sequence positions are iteratively and randomly mutated and mutations are accepted if the objective function improves. In the case of RNAinverse, the objective function reflects the Hamming distance between the predicted *minimum free energy* (MFE) structure of the design candidate and the target secondary structure. The optimization procedure stops if and when the Hamming distance reaches zero. We note that there is no guarantee for the optimization procedure to find an optimal solution and therefore it is required to specify a cap for the maximum number of iterations allowed.

Subsequent RNA designer methods have demonstrated improved performance compared to RNAinverse. RNA-SSD (Andronescu et al., 2004) and INFO-RNA (Busch and Backofen, 2006) introduced improved seed initialization techniques and stochastic local search strategies to design RNAs with high thermostability. NUPACK (Zadeh et al., 2011) introduced a weighted local sampling strategy to design RNA sequences with low ensemble defect. RNAexinv (Avihoo et al., 2011) used a multi-objective optimization strategy to design RNAs with high thermostability and high mutational robustness. RNAensign (Levin et al., 2012) took a global sampling strategy to design RNAs with high thermostability. Frnakenstein (Lyngsø et al., 2012) utilized a genetic algorithm with local sampling strategy to design RNAs for multiple target structures. RNAiFold (Garcia-Martin et al., 2013) defines the sequence design as a constraint satisfaction problem to design RNAs with targeted GC content and high thermostability. IncaRNAtion (Reinharz et al., 2013) introduces a glocal sampling strategy to design RNAs with targeted GC content and high thermostability.

All above mentioned RNA designer methods ignore a critical structural element called pseudoknot and therefore have limited use. A pseudoknot is typically formed when crossing basepairs occur between the unpaired bases from a loop and other bases outside that loop. Several ncRNA species with regulatory function such as glmS ribozymes (Klein and Ferré-D'Amaré, 2006; Soukup, 2006), Delta ribozymes (Nehdi et al., 2007), SAM II aptamer domain (Gilbert et al., 2008), SAH riboswitch aptamer domain (Edwards et al., 2010), Hammerhead riboyzmes (Perreault et al., 2011) and Twister ribozymes (Roth et al., 2014) contain pseudoknots, where the pseudoknots are known to stabilize the functional form of the structure. Hence, it is of interest to develop RNA designer methods that can handle pseudoknots as well. Computational complexity of designing pseduoknotted RNA secondary structures is characterized by Ponty and Saule (2011).

We identify three reasons why the above mentioned methods can not handle the design of pseudoknotted RNAs. First, in all of the above methods the folding algorithms used to predict the folding properties of the designed sequences are often RNAfold or mfold. Even though both RNAfold and mfold can predict the MFE structure and the *partition function* (McCaskill, 1989) of a given sequence and a given target structure of length *n* in *O*(*n*^3^) time and *O*(*n*^2^) space, neither can be used to predict presence of pseudoknots. Second, all above mentioned methods utilize hierarchical structural decomposition methods to speed up the design process. However, the hierarchical structural decomposition methods used by the previous methods can not be generalized to cover pseudoknots and therefore are inapplicable. Third, none of the above methods make any distinction between the different types of base pairs (i.e., nested vs. non-nested) and therefore are not well suited for the cases where the secondary structure includes a pseudoknot motif. In order to include pseudoknots in the design process, it is crucial to address the above mentioned issues.

To our knowledge, there are three algorithmic reports in the literature for the design of pseudoknotted RNAs. antaRNA (Kleinkauf et al., 2015) utilizes an Ant Colony Optimization technique (Dorigo et al., 2006) to design pseudoknotted RNAs that are predicted to fold into the target structure with targeted GC distribution. antaRNA (Kleinkauf et al., 2015) uses pKiss (Janssen and Giegerich, 2015) to predict the MFE structure of the RNA sequences including pseudoknots. MODENA (Taneda, 2012) is a multi-objective genetic algorithm (MOGA) for pseudoknotted RNA sequence design. MODENA attempts to maximize the structural similarity between the target structure and the predicted fold while simultaneously minimizing the free energy of the design candidate sequences. MODENA implements a novel crossover operator to handle pseudoknots and uses IPknot (Sato et al., 2011) as its default folding algorithm. For a given RNA sequence, IPknot can predict the pseudoknotted secondary structure with *maximum expected accuracy* (MEA) (Lu et al., 2009); hence enabling MODENA to design pseudoknotted RNAs. Note that neither IPknot nor pKiss can not compute the partition function and therefore can not be used to measure important qualitative characteristics such as the *ensemble defect* and the *probability defect* of the sequences. The term ensemble defect corresponds the ensemble average of the incorrectly pair nucleotides and the term probability defect corresponds to the sum of the probabilities of all non-target structures in the structural ensemble at thermodynamic equilibrium (Zadeh et al., 2011). INV (Gao et al., 2010) is another RNA designer algorithm to design a restricted class of pseudoknots using a graph decomposition method and a energy minimization criteria. However, as reported by Taneda (2012), the current implementation of INV, does not return any solution for structures larger than 85 nucleotides. It is also worth noting that the benchmark data set of the original article for INV, contains only four structures that are all shorter than 85 nucleotides in length.

In our work, we identify three key choices for the design of pseudoknotted RNAs and devise a new sequence design algorithm. First is the choice for the folding algorithm, which must recognize pseudoknots. Ideally one requires the folding algorithm to compute two key measures: (i) the free energy of the folded molecule, and (ii) the partition function of a single RNA sequence when folded into a target pseudoknotted secondary structure. The free energy is a measure of thermostability, and the partition function makes it possible to characterize the equilibrium base pair qualities by computing the matrix of base pair probabilities. Most of the widely used single sequence folding algorithms such as RNAfold and mfold can not characterize pseudoknots. On the other hand, other existing methods, which can recognize the pseudoknots such as IPknot, Hotknot (Ren et al., 2005), ProbKnot (Bellaousov and Mathews, 2010), pKiss and NanoFolder (Bindewald et al., 2011), can only compute the free energy of the pseudoknotted structures and do not make it possible to compute the partition function. To our knowledge, NUPACK is the only available method, which can be utilized to compute the partition function of a limited but biologically relevant class of pseudoknots (Dirks and Pierce, 2003) and therefore make it possible to compute the matrix of base pair probabilities of a single sequence folding into pseudoknotted target structures. Using the matrix of base pair probabilities, one can compute two other important measures namely ensemble defect and probability defect as well.

The second sequence design choice is the choice an objective function for the optimization algorithm. antaRNA, MODENA and INV utilize energy minimization approaches to design RNA sequences that have the highest similarity to the target structure by favoring design candidates that have lower free energy when folded into the target. However, as described and demonstrated by Dirks et al. (2004) and Zadeh et al. (2011), ensemble defect optimization dominates both of the energy minimization and probability defect minimization approaches. More precisely, ensemble defect minimization leads to design of molecules with folding energies that are as low as those of the molecules designed by energy minimization approaches and also have probability defect values that are as low as those of the molecules designed through probability defect minimization methods. Hence, the ideal choice for the objective function would be the ensemble defect minimization and (Zadeh et al., 2011) provides sufficient evidence to support this claim.

The third sequence design choice is an efficient search strategy which may be realized via iterative sequence mutations. It is desirable for the mutation operators to be able to make distinction between different types of base pairs (i.e., nested base pairs and non-nested base pairs), while efficiently exploring the mutational landscape of the design candidates. To efficiently explore the mutational landscape of the design candidates, the mutation operator must make effective use of the folding attributes, such as the free energy as well as the two different matrices of base pair probabilities, as predicted by the folding algorithm.

In this paper, we follow an ensemble defect optimization strategy to design RNA sequences that fold into a single targeted secondary structure that include pseudoknots. Our method extends the approach previously introduced by Zadeh et al. (2011) to design pseudoknot-free RNA secondary structures such that the pseudoknots can be handled as well. We introduce a new *adaptive defect weighted sampling* algorithm named Enzymer, and use it to progressively mutate design candidates until the specified stop conditions are reached. We note that the notion of adaptive weighted sampling technique was previously used by Reinharz et al. (2013) in another context. To benchmark our method, we used a biological dataset from the PseudoBase library (Van Batenburg et al., 2000), containing 201 pseudoknotted ncRNAs of length 21–140 nucleotides. We compared our results with the results generated by the state of the art namely MODENA and antaRNA. The data shows that the population of the sequences generated by Enzymer have lower ensemble defect, lower probability defect, higher Boltzmann frequency and higher success rate when compared to MODENA. Our results also show that Enzymer generates sequence populations that have lower ensemble defect, lower probability defect, higher thermostability, higher Boltzmann frequency and higher success rate when compared to the results generated by antaRNA. Finally, we used Enzymer and constrained the design process by using a naturally occurring and highly conserved Hammerhead motif and designed 8 RNA sequences for a pseudoknotted *cis*-acting Hammerhead ribozyme.

## 2. Materials and methods

### 2.1. RNA folding measures at equilibrium

Let ϕ denote an RNA sequence with *n* nucleotides. Sequence ϕ = ϕ_1_ … ϕ_*n*_, can be specified by positional base identities such that ϕ_*i*_ ∈ {*A, U, G, C*} for *i* = 1, …, *n*. Secondary structure τ can be specified by a set of base pairs (ϕ_*i*_, ϕ_*j*_) where 1 ≤ *i* < *j* ≤ *n*, such that positions *i* and *j* are paired, *j* ≥ *i* + 3, and (ϕ_*i*_, ϕ_*j*_) ∈ {(*A* − *U*), (*G* − *C*), (*G* − *U*), (*U* − *A*), (*C* − *G*), (*U* − *G*)}. We denote ensemble Γ, as the set of all possible secondary structures of ϕ including pseudoknots. For a sequence ϕ and secondary structure τ ∈ Γ, the *free energy* Δ*G*(ϕ, τ) in kcal/mol, is calculated using nearest-neighbor empirical parameters for RNA in 1 M *Na*^+^ (Mathews et al., 1999). By calculating the *partition function* (Dirks and Pierce, 2003) over Γ:

(1) $$\begin{array}{l} Q\left(\phi \right)=\underset{\tau \in \Gamma }{\sum }e^{-\Delta G\left(\phi ,\tau \right)∕k_{B}T} \end{array}$$

one can evaluate the *equilibrium probability* of ϕ folding into τ:

(2) $$\begin{array}{l} p\left(\phi ,\tau \right)=\frac{1}{Q\left(\phi \right)}e^{-\Delta G\left(\phi ,\tau \right)∕k_{B}T} \end{array}$$

where *k*_*B*_ is the Boltzmann constant and *T* is the temperature in Kelvin. The equilibrium structural features of ensemble Γ are quantified by the *base pairing probability matrix P*(ϕ) with entries *P*_*i, j*_ ∈ [0, 1] corresponding to the probability:

(3) $$\begin{array}{l} P_{i,j}\left(\phi \right)=\underset{\tau \in \Gamma }{\sum }p\left(\phi ,\tau \right)S_{i,j}\left(\tau \right) \end{array}$$

that the base pair *i*.*j* forms at equilibrium. Here *S*(τ) is the *structure matrix* with entries *S*_*i, j*_ ∈ {0, 1}. If structure τ contains pair *i*.*j*, then *S*_*i, j*_ = 1, otherwise *S*_*i, j*_ = 0. To describe unpaired bases, the structure and probability matrices are augmented by an extra column. The entry *S*_*i, n*+1_(τ) is unity if base *i* is unpaired in structure τ and zero otherwise. The entry *P*_*i, n*+1_(ϕ) ∈ [0, 1] denotes the equilibrium probability that base *i* is unpaired over ensemble Γ. Hence, the row sums of the augmented *S*(τ) and *P*(ϕ) are unity. The term *probability defect* (Zadeh et al., 2011) corresponding to the sum of the probabilities of all non-target structures of ensemble Γ can be computed by term:

(4) $$\begin{array}{l} \pi \left(\phi ,\tau \right)=1-p\left(\phi ,\tau \right) \end{array}$$

The term *ensemble defect* (Zadeh et al., 2011) is defined by:

(5) $$\begin{array}{l} n\left(\phi ,\tau \right)=n-\underset{1\leq i\leq n,1\leq j\leq n+1}{\sum }P_{i,j}\left(\phi \right)S_{i,j}\left(\tau \right) \end{array}$$

where *n*(ϕ, τ) corresponds to the ensemble average number of incorrectly paired nucleotides at equilibrium over ensemble Γ. Intuitively, the term *normalized ensemble defect* is given by:

(6) $$\begin{array}{l} N\left(\phi ,\tau \right)=n\left(\phi ,\tau \right)∕n \end{array}$$

We use NUPACK to compute *P*_*i, j*_ as well as two extra matrices: the matrix of nested base-pair probabilities $P_{i,j}^{\prime }$, and the matrix of non-nested base-pair probabilities $P_{i,j}^{\prime \prime }$, all in *O*(*n*^5^) time and *O*(*n*^4^) space. The dynamic programming methods to compute $P_{i,j}^{\prime }$ and $P_{i,j}^{\prime \prime }$ are described by Dirks and Pierce (2003). Enzymer uses *P*_*i, j*_ to compute the normalized ensemble defect, and uses $P_{i,j}^{\prime }$ and $P_{i,j}^{\prime \prime }$ to guide the mutation operator.

One can formulate the *MFE defect* by term:

(7) $$\begin{array}{l} \mu \left(\phi ,\tau \right)=d\left(MFE_{\phi },\tau \right) \end{array}$$

where *d*(*MFE*_ϕ_, τ) quantifies the hamming distance between the predicted MFE structure of ϕ and the target structure τ. We call a design *successful* if *d*(*MFE*_ϕ_, τ) = 0. Furthermore, to measure how dominant a structure is in the Boltzmann ensemble, one can compute the Boltzmann frequency by term:

(8) $$\begin{array}{l} B_{f}=e^{-\Delta G\left(\phi ,\tau \right)∕k_{B}T}∕Q\left(\phi \right) \end{array}$$

Finally, for a set of aligned sequences $S=\left\{\phi^{1}\ldots \phi^{l}\right\}$ generated for a single target τ, the term *sequence identity* (Reinharz et al., 2013) defined by:

(9) $$\begin{array}{l} S_{id}=\underset{\phi^{1},\phi^{2}\in S\times S}{\sum }\left(\frac{1}{\phi^{1}}\underset{\phi_{i}^{1}\equiv \phi_{i}^{2}}{\sum }1\right) \end{array}$$

quantifies the the degree of similarity of the sequences in the corresponding set *S*. Intuitively, *S*_*id*_ quantifies the diversity of a sequence population. Note that in our case all sequences designed for a given structure have equal length and therefore there are no gaps in the aligned set *S*.

### 2.2. Adaptive defect weighted sampling algorithm

Enzymer follows an ensemble defect minimization approach and implements a new *adaptive defect weighted sampling* algorithm to design pseudoknotted RNAs with a single target secondary structure. In our context, the term *adaptive* means that the total number of positions to mutate at each iteration, is dynamically chosen at the run-time. The term *defect weighted sampling* means that at each iteration the probability of mutation of a nucleotide at each position depends on the type of that position (i.e., free, nested pair or non-nested pair), and is also proportional to the positional contribution of that nucleotide to the ensemble defect of the sequence. The positional defect of each position is based on the type of the position and is quantified by *P*_*i, j*_ for free nucleotides, by $P_{i,j}^{\prime }$ for nested base pairs, and by $P_{i,j}^{\prime \prime }$ for non-nested base pairs.

For a given pseudoknotted target structure τ of size *n*, our method starts with a randomly generated seed ϕ, and iteratively samples from the low ensemble defect mutational landscape of the seed until it reaches the stop condition. Let *f*_*stop*_ denote the maximum value that we accept for *N*(ϕ, τ). The iterations stop and return ϕ when *N*(ϕ, τ) ≤ *f*_*stop*_. We note that during each instance of the design trial, there is no guarantee of reaching *N*(ϕ, τ) ≤ *f*_*stop*_. Hence, we limit the maximum number of the iterations and once the limit is reached, we report the fittest result that was found during the sampling process. Let *max*_*it* denote the maximum number of iterations. Then we define the *stop condition* as the event where either *N*(ϕ, τ) ≤ *f*_*stop*_ or *max*_*it* is reached.

**Figure 1:**
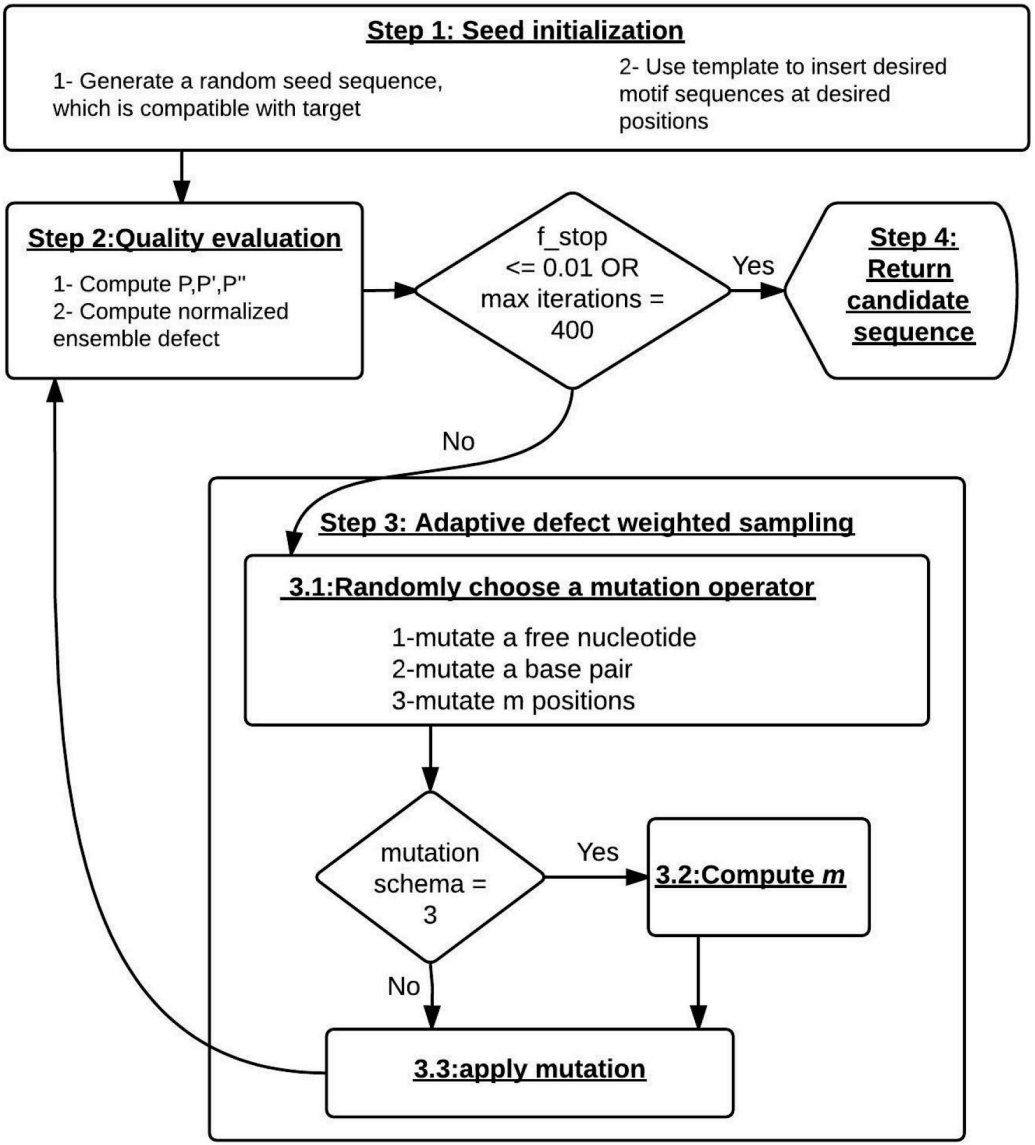
**The design pipeline of Enzymer**. Step 1: we generate a random seed sequence, which is compatible with the target. Step 2: we evaluate the quality of the sequence. If the of the stop condition is met, we return the sequence. Step 3: the adaptive defected weighted sampling process starts here. In 3.1 the mutation operator is uniformly randomly selected. If the *m-mutation* schema is chosen. In step 3.2 we compute the value of *m*. In 3.3 we sample from low ensemble defect mutational landscape of the current sequence by applying the mutation operator. Step 4: when the stop condition is reached, we return the designed sequence.

Figure 1 presents the key steps of Enzymer. Algorithm 1 describes the complete design approach. Algorithms 2–4, describe the three mutation operators that constitute the adaptive defect weighted sampling process. An Enzymer instance, starts with four input parameters: (i) τ, (ii) *f*_*stop*_, (iii) *max*_*it*, and (iv) design template *t* as defined by string *t* = *t*_1_ … *t*_*n*_, where *t*_*i*_ ∈ {*A, U, G, C, o*} such that the length of *t* is equal to *n*. We use *t* to specify design constrains.

**Algorithm 1:** *Enzymer*(τ, *f*_*stop*_, *max*_*it*, *t*, 3)

1: // input: target structure, target normalized ensemble defect, maximum iterations and the design template
2: ϕ ← *initialize_seed*(τ, *t*)
3: *iteration_count* ← 1
4: *C*_*design_begin*_ ← *current_time*()
5: $P_{i,j},P_{i,j}^{\prime },P_{i,j}^{\prime \prime },\pi \left(\phi ,\tau \right)\leftarrow nupack\text{\_}pairs\left(\phi ,\tau \right)$ //compute pair probabilities using NUPACK-pairs
6: *N*(ϕ, τ) ← *compute_normalized_ensemble_defect*(*P*_*i, j*_, ϕ, τ)
7: // adaptive defect weighted sampling process starts here
8: **while** (*N*(ϕ, τ) ≥ *f*_*stop*_) OR (*iteration_count* < *max_it*) **do**
9: *iteration_count* ← *iteration_count*+1
10: *mutation_scheme* ← *random_integer*(1, 3)
11: **if** (*mutation_scheme* = = 1) **then**
12: ϕ ← *mutate_single_nucleotide*(ϕ, τ, *P*_*i, j*_, *t*)
13: **end if**
14: **if** (*mutation_scheme* = = 2) **then**
15: $\phi \leftarrow mutate\text{\_}basepair\left(\phi ,\tau ,P_{i,j}^{\prime },P_{i,j}^{\prime \prime },t\right)$
16: **end if**
17: **if** (*mutation_scheme* = = 3) **then**
18: *m*′ ← (*length*(τ)*N(ϕ, τ))∕5
19: *m* ← *floor*(*absolute*_*value*(*normal*_*distribution*(*m*′, *m*′∕5)))
20: **if** *m* < 1 **then**
21: *m* = 1
22: **end if**
23: $\phi \leftarrow m\text{\_}mutants\left(m,\phi ,\tau ,P_{i,j},P_{i,j}^{\prime },P_{i,j}^{\prime \prime },t\right)$
24: **end if**
25: $P_{i,j},P_{i,j}^{\prime },P_{i,j}^{\prime \prime },\pi \left(\phi ,\tau \right)\leftarrow nupack\text{\_}pairs\left(\phi ,\tau \right)$
26: *N*(ϕ, τ) ← *compute_normalized_ensemble_defect*(*P*_*i, j*_, ϕ, τ)
27: **end while**
28: *C*_*design_end*_ ← *current_time*()
29: *C*_*design*_ ← *C*_*design_end*_ − *C*_*design_begin*_
30: Return ϕ, *N*(ϕ, τ), π(ϕ, τ), Δ*G*(ϕ, τ), *C*_*design*_

**Algorithm 2:** *mutate_single_nucleotide*(ϕ, τ, *P*_*i, j*_, *t*)

1: // input: sequence, target structure, matrix of pair probabilities and the design template
2: *mutation* ← *False*
3: **while** *mutation* = = *False* **do**
4: *i* ← *randomly_select_unpaired_position*(τ)
5: **if** *t*[*i*] is not “*o*” **then**
6: continue
7: **end if**
8: *random_number* ← *random_float*(0, 1)
9: *probability_of_mutation* ← 1−*P*_*i, n*+1_
10: **if** *random_number* < *probability_of_mutation* **then**
11: ϕ′ ← *mutate*_*at*_*position*(*position* = *i*, ϕ) //replace ϕ_*i*_ with A,G,U or C
12: **if** ϕ′ is not ϕ **then**
13: ϕ ← ϕ′
14: *mutation* ← *True*
15: **end if**
16: **end if**
17: **end while**
18: Return ϕ

**Algorithm 3:** $mutate\text{\_}basepair\left(\phi ,\tau ,P_{i,j}^{\prime },P_{i,j}^{\prime \prime },t\right)$

1: //function inputs: sequences, target, nested pair probability, non-nested pair probability, template
2: *mutation* ← *False*
3: **while** *mutation* = = *False* **do**
4: *i, j* ← *randomly*_*select*_*a*_*pair*(τ)
5: **if** *t* [*i*] is not “*o*” AND *t* [*j*] is not “*o*” **then**
6: continue // The entire pair is locked as specified by the design template *t*
7: **end if**
8: **if** *t* [*i*] is not “*o*” AND *t* [*j*] is “*o*” **then**
9: ϕ′ ← *only*_*mutate*_*j*(*j*, ϕ) // respecting pair rules, only mutate the unlocked part of the pair
10: **if** ϕ′ is not ϕ **then**
11: ϕ ← ϕ′
12: *mutation* ← *True*
13: **end if**
14: Return ϕ
15: **end if**
16: **if** *t* [*j*] is not “*o*” AND *t* [*i*] is “*o*” **then**
17: ϕ′ ← *only*_*mutate*_*i*(*i*, ϕ) // respecting pair rules, only mutate the unlocked part of the pair
18: **if** ϕ′ is not ϕ **then**
19: ϕ ← ϕ′
20: *mutation* ← *True*
21: **end if**
22: Return ϕ
23: **end if**
24: **if** (*i, j*) is a nested base pair in τ **then**
25: *random*_*number* ← *random*_*float*(0, 1)
26: $probability\text{\_}of\text{\_}mutation\leftarrow 1-P_{i,j}^{\prime }$
27: **if** *random*_*number* < *probability*_*of*_*mutation* **then**
28: ϕ′ ← *mutate*_*position*_*i*_*j*(ϕ, *i, j*) //replace ϕ_*i*_, ϕ_*j*_ with A-U, G-C or G-U
29: **if** ϕ′ is not ϕ **then**
30: ϕ ← ϕ′
31: *mutation* ← *True*
32: **end if**
33: **end if**
34: **end if**
35: **if** (*i, j*) is a non-nested base pair in τ **then**
36: *random*_*number* ← *random*_*float*(0, 1)
37: $probability\text{\_}of\text{\_}mutation\leftarrow 1-P_{i,j}^{\prime \prime }$
38: **if** *random*_*number* < *probability*_*of*_*mutation* **then**
39: ϕ′ ← *mutate*_*position*_*i*_*j*(ϕ, *i, j*) //replace ϕ_*i*_, ϕ_*j*_ with A-U,G-C,G-U,U-A,C-G or U-G
40: **if** ϕ′ is not ϕ **then**
41: ϕ ← ϕ′
42: *mutation* ← *True*
43: **end if**
44: **end if**
45: **end if**
46: **end while**
47: Return ϕ

**Algorithm 4:** $m\text{\_}mutation\left(m,\phi ,\tau ,P_{i,j},P_{i,j}^{\prime },P_{i,j}^{\prime \prime },t\right)$

1: // This function mutates exactly *m* positions. The inputs are the number of positions to mutate, sequence, target structure, pair probabilities, nested pair probabilities, non-nested pair probabilities and the design template, respectively.
2: *mutation*_*count* ← 0
3: **while** *mutation*_*count* < *m* **do**
4: *i* ← *random*(1, *length*(τ))
5: **if** ϕ_*i*_ is a free nucleotide OR *mutation*_*count* = = (*m* − 1) **then**
6: ϕ ← *mutate*_*single*_*nucleotide*(ϕ, τ, *P*_*i, j*_, *t*)
7: *mutation*_*count* ← *mutation*_*count* + 1
8: **end if**
9: **if** ϕ_*i*_ is not a single nucleotide **then**
10: $\phi \leftarrow mutate\text{\_}basepair\left(\phi ,\tau ,P_{i,j}^{\prime },P_{i,j}^{\prime \prime },t\right)$
11: *mutation*_*count* ← *mutation*_*count* + 2
12: **end if**
13: **end while**
14: Return ϕ

First, for target τ we initialize a random RNA seed sequence ϕ that is compatible with the target structure by enforcing base pairing rules (Algorithm 1, line 2). At the seed initialization step, the design template *t* is used as a mean to specify a set of positional nucleotide constrains on the seed sequence. Once the seed is initialized, we update the seed to match the template such that for *i* = 1…*n*, if *t*_*i*_ ≠ ”*o*” then ϕ_*i*_ = *t*_*i*_. Furthermore, *t* is also used during the sampling process to safeguard the constrained positions against mutations. More precisely, for *i* = 1…*n*, the nucleotide ϕ_*i*_ is subject to mutation, if and only if *t*_*i*_ = ”*o*”. Our algorithm allows the user to specify the percentage of the GC content for unconstrained regions of the initial seed sequence and if the GC content is not specified, a random value between of 20 and 80% is used to generate the initial seed sequence.

Second, we use the prob program with -pseudo option from NUPACK, to compute *P*_*i, j*_, $P_{i,j}^{\prime }$ and $P_{i,j}^{\prime \prime }$. We use *P*_*i, j*_ to compute *N*(ϕ, τ) and use $P_{i,j}^{\prime }$ and $P_{i,j}^{\prime \prime }$ to guide the sampling step (Algorithm 1, lines 5 and 6).

Third, the algorithm executes the adaptive defect weighted sampling process until it reaches the stop condition (Algorithm 1, line 8). At each iteration the sampling process will uniformly and randomly select from one of the mutation operator (Algorithm 1, line 10) to sample mutants from the low ensemble defect mutational landscape of ϕ. The first mutation operator targets unpaired positions and mutates a single unpaired position. The second mutation operator targets pair positions and mutates a single base pair. Ideally, we would like to mutate multiple positions at each iteration with the aim of reaching the stop criteria with fewer iterations and therefore reducing the running time of the sampling algorithm. Therefore we implemented a third mutation operator to dynamically decide for variable *m*, which quantifies the total number of positions that have to go under mutation at each iteration. Once the third mutation operator computes *m* it will make random calls to the first and second mutation operators until precisely *m* positions are mutated. The details of each of the three mutation operators follows:

1. **single point mutation** (algorithm 2): this operator samples a mutant sequence from the mutational landscape of ϕ by mutating a single free nucleotide. For an arbitrary unpaired ϕ_*i*_, the probability of mutation is computed by (1 − *P*_*i, n*+1_), which is the measure of positional contribution of ϕ_*i*_ to *N*(ϕ, τ). The mutation operator scans through ϕ until it selects a single unpaired nucleotide ϕ_*i*_ for mutation.
2. **pair mutation** (algorithm 3): this operator samples a mutant sequence from the mutational landscape of ϕ by mutating a single base pair. This operator makes distinction between the two different types of base pairs. For an arbitrary nested base pair (ϕ_*i*_, ϕ_*j*_), the probability of pair mutation is proportional to its contribution to *N*(ϕ, τ) and is computed by the term $\left(1-P_{i,j}^{\prime }\right)$. For an arbitrary non-nested base pair (ϕ_*i*_, ϕ_*j*_), the probability of pair mutation is proportional to its contribution to *N*(ϕ, τ) and is computed by $\left(1-P_{i,j}^{\prime \prime }\right)$. The operator continuously scans through all base pairs to select exactly one base pair for mutation.
3. **m-mutation** (algorithm 4): this operator samples a mutant sequence from the mutational landscape of ϕ by mutating exactly *m* positions. The value of *m* will dynamically converge to a value proportional to *N*(ϕ, τ) and *n*. Let *m*′ represent the value that *m* converges to and be defined by:
  (10) $$\begin{array}{l} m^{\prime }=\left(N\left(\phi ,\tau \right)*n\right)∕C \end{array}$$
  where *C* is an arbitrary constant. In our simulations we set *C* = 5. Then we compute *m* using:
  (11) $$m=\left\lfloor \left|normal\_distribution(m^{\prime },m^{\prime }/5)\right|\right\rfloor$$
  Once the value of *m* is determined, the operator will iteratively make uniformly random calls to the single point and pair mutation operators until exactly *m* positions are mutated. This technique causes the sampling process to choose more positions for mutation when *N*(ϕ, τ) is large, and to choose fewer positions as *N*(ϕ, τ) diminishes.

The last step of each iteration is to compute $N\left(\phi ,\tau \right),P_{i,j},P_{i,j}^{\prime },P_{i,j}^{\prime \prime }$ and to decide whether the stop condition is reached or not. Finally, when the sampling process reaches the stop condition, the iterations will stop and ϕ will be returned.

### 2.3. Characterizing performance of the optimization algorithm

To measure the run-time performance of each Enzymer instance, we count the number of iterations as well as the number of seconds that were required to reach the stop criteria. We emphasize that our algorithm utilizes NUPACK to compute the partition function of each sequence in *O*(*n*^5^) time. Due to the expensive computational costs associated with computation of the partition function at each iteration, it would be ideal to utilize an approach that enables the algorithm to reach the stop criteria in fewer steps. We will discuss in the results section how our third mutation operator (i.e., *m* − *mutation* operator) improves the run-time requirement of our adaptive weighted sampling algorithm.

### 2.4. Dataset

To benchmark the performance of our method we use a non-redundant and diverse biological dataset of pseudoknotted secondary structures prepared by Taneda (2012). We note that the original source of all the target structures in this dataset is the Pseudobase library. The initial dataset was composed of 266 structures. We emphasize that the only existing folding algorithm which enables one to compute *P*(ϕ, τ), *P*′(ϕ, τ) and *P*″(ϕ, τ), is NUPACK and therefore we use it to filter the dataset. Since NUPACK can only recognize a limited class of pseudoknots, our filtering process yields a dataset of 201 pseudoknotted structures of length 21–140 nucleotides. Figure 1 in the Supplementary Material section presents the size distribution of the target structures in the filtered dataset. We will refer to the filtered dataset as Pseudo. Our algorithm accepts secondary structures over the alphabet {[, ], (, ), .} presented in standard dot bracket notation. The Pseudo dataset is available at https://bitbucket.org/casraz/enzymer.

## 3. Results

### 3.1. Setup

For each target structure in Pseudo, we ran Enzyner for 30 independent trials. We ran each trial on a dedicated computational core with a CPU speed of 2.0 GHz and 2 GB of RAM. This leads to 30*201 (total of 6030) independent instances of the method. In our setup, we set *f*_*stop*_ = 0.01 and *max*_*it* = 400. Note *max*_*it* = 400 is an arbitrary choice; however as we will discuss, it turned out the 400 is a sufficiently large number of iterations to demonstrate the effectiveness of our approach. Finally, Enzymer returns a single design candidate per trial.

We compare the performance of Enzymer with MODENA and antaRNA. We emphasize that for target structure τ, Enzymer seeks to design sequence ϕ by minimizing the normalized ensemble defect value, where MODENA and antaRNA aim to design sequences with high thermostability. In order to establish a fair basis for comparison with MODENA, we set the maximum number of generations (i.e., *max*_*it*) of a MODENA instance to 400. Note that MODENA is a genetic algorithm and is initialized by a population of *P* independently generated seed sequences and once it reaches the maximum number of generations it returns a population of *P* candidate solutions. In order to observe a consistent behavior, the author of MODENA (Taneda, 2012) recommends to set the initial population size to be equal to 10% of the total number of generations. Hence, for each target structure we set the *P* = 40. In the end, for each target structure, we sort the generated sequences based on the corresponding normalized ensemble defect values and select a subset of 30 sequences with the lowest normalized ensemble defect. MODENA generated sequences for all of the 201 target structures. For the case of antaRNA, we ran 30 independent trials and generated 30 sequences for each target structure. Because there is no guarantee that antaRNA reaches the stop condition, we limit the running time to be equal median running time that was required by Enzymer to reach the stop condition for each corresponding target structure. We note that antaRNA failed to recognize 4 of the target structures from the benchmark dataset.

Other than MODENA and antaRNA, the only other reported pseudoknot designer algorithm is INV. As of the date of submission of this article, INV has remained unavailable for benchmarking purposes. However, as reported by Taneda (2012), INV does not return any solution for structures that are larger than 85 nucleotides in length. Furthermore, even for structures that are shorter than 85 nucleotides, MODENA has demonstrated superior performance compared to INV. Therefore comparing Enzymer with MODENA and antaRNA is expected to provide us with sufficient information about the performance of Enzymer.

### 3.2. Benchmark results

To characterize the quality of a designed sequence ϕ that is predicted to fold into τ, we measure the normalized ensemble defect *N*(ϕ, τ) (Equation 6), probability defect π(ϕ, τ) (Equation 4), normalized free energy Δ*G*(ϕ, τ), MFE defect μ(ϕ, τ) (Equation 7), Boltzmann frequency *B*_*f*_ (Equation 8) and sequence identity *S*_*id*_ (Equation 9).

**Figure 2:**
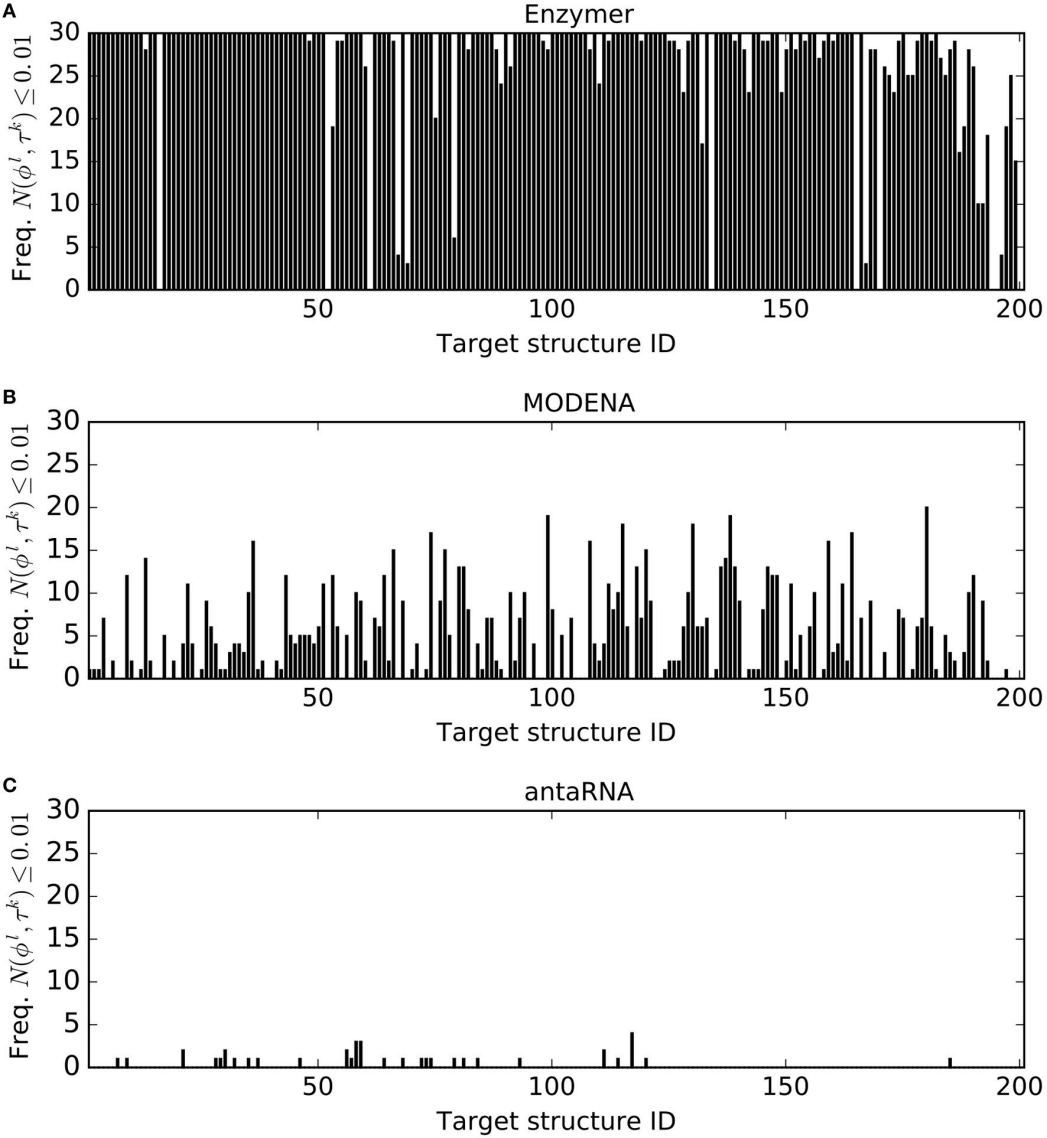
**Frequency of the solutions per structure where *N*(ϕ^*l*^, τ^*k*^) ≤ 0.01**. For each target τ^*k*^ ∈ Pseudo for *k* = 1…201, the corresponding vertical bar represents the frequency (out of 30 trials) of the generated sequences ϕ^*l*^ for *l* = 1…30, where *N*(ϕ^*l*^, τ^*k*^) ≤ 0.01. **(A)** Enzymer generated at least one sequence ϕ^*l*^ such that *N*(ϕ^*l*^, τ^*k*^) ≤ 0.01 for 188 of the structures. **(B)** MODENA generated at least one sequence ϕ^*l*^ such that *N*(ϕ^*l*^, τ^*k*^) ≤ 0.01 for 144 of the structures. **(C)** antaRNA generated at least one sequence ϕ^*l*^ such that *N*(ϕ^*l*^, τ^*k*^) ≤ 0.01 for 24 of the structures. Binomial statistic test with 99% confidence, suggests Enzymer significantly outperforms both MODENA and antaRNA in generating sequences such that *N*(ϕ^*l*^, τ^*k*^) ≤ 0.01. Notably, the binomial test also suggests superior performance of MODENA compared with antaRNA. Structure IDs on the x-axis are sorted based on increasing size of the corresponding targets.

For each of the three methods and for each target structure τ^*k*^ ∈ Pseudo where *k* = 1, …, 201, we generated 30 sequences ϕ^*l*^s where *l* = 1, …, 30. For each τ^*k*^, let *f*^*k*^ denote the frequency of reaching *N*(ϕ^*l*^, τ^*k*^) ≤ 0.01. Figure 2 presents the *f*^*k*^ values we obtained for each τ^*k*^ from a pool of 30 generated ϕ^*l*^ by each method. In this performance evaluation, we observed *f*^*k*^ ≥ 1 in 188, 144, and 24 cases for Enzymer (Figure 2A), for MODENA (Figure 2B) and for antaRNA (Figure 2C) respectively. Furthermore, we observed that there is no single case where the *f*^*k*^ of the results generated by Enzymer was lower than that of MODENA or antaRNA.

**Figure 3:**
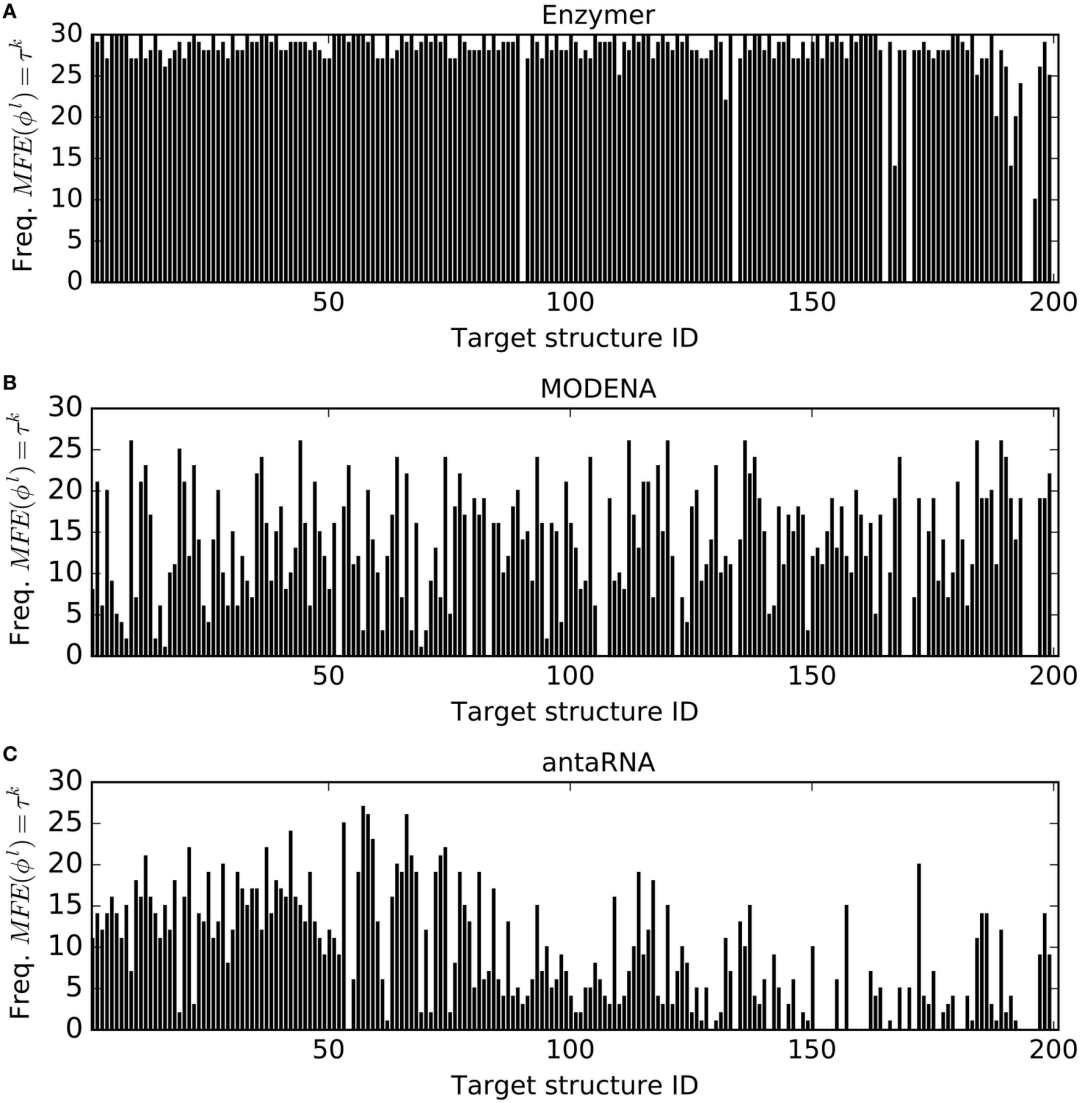
**MFE defect**. For each target τ^*k*^ ∈ Pseudo for *k* = 1…201, the corresponding vertical bar represents the frequency (out of 30 trials) where *MFE*(ϕ^*l*^, τ^*k*^) = τ^*k*^ was reached. Comparison of performance of Enzymer **(A)** with the performance of MODENA **(B)** and antaRNA **(C)** shows Enzymer outperformed the other two methods in 191 and 194 cases respectively. A binomial test statistic with 99% confidence suggests Enzymer outperforms both methods in generating sequences with lower MFE defect. Furthermore, MODENA outperforms antaRNA in 127 cases and the binomial test statistic suggests superior performance of MODENA compared with antaRNA. Structure IDs on the x-axis are sorted based on increasing size of the corresponding targets.

The number of successful designs where μ(ϕ^*l*^, τ^*k*^) = 0 are presented in Figure 3. The results show Enzymer outperformed MODENA and antaRNA in 191 and 194 cases respectively. We also observe MODENA outperformed antaRNA in 127 cases. Respective binomial test statistics with *p*-values 1.55*e*^−44^ and 1.52*e*^−48^ suggest Enzymer delivers superior performance compared to MODENA and antaRNA in generating sequences that have their predicted MFE equal to the target structure. Moreover, binomial test statistic with *p*-value 2.26*e*^−4^ also suggests that MODENA delivers superior performance compared to antaRNA.

**Figure 4:**
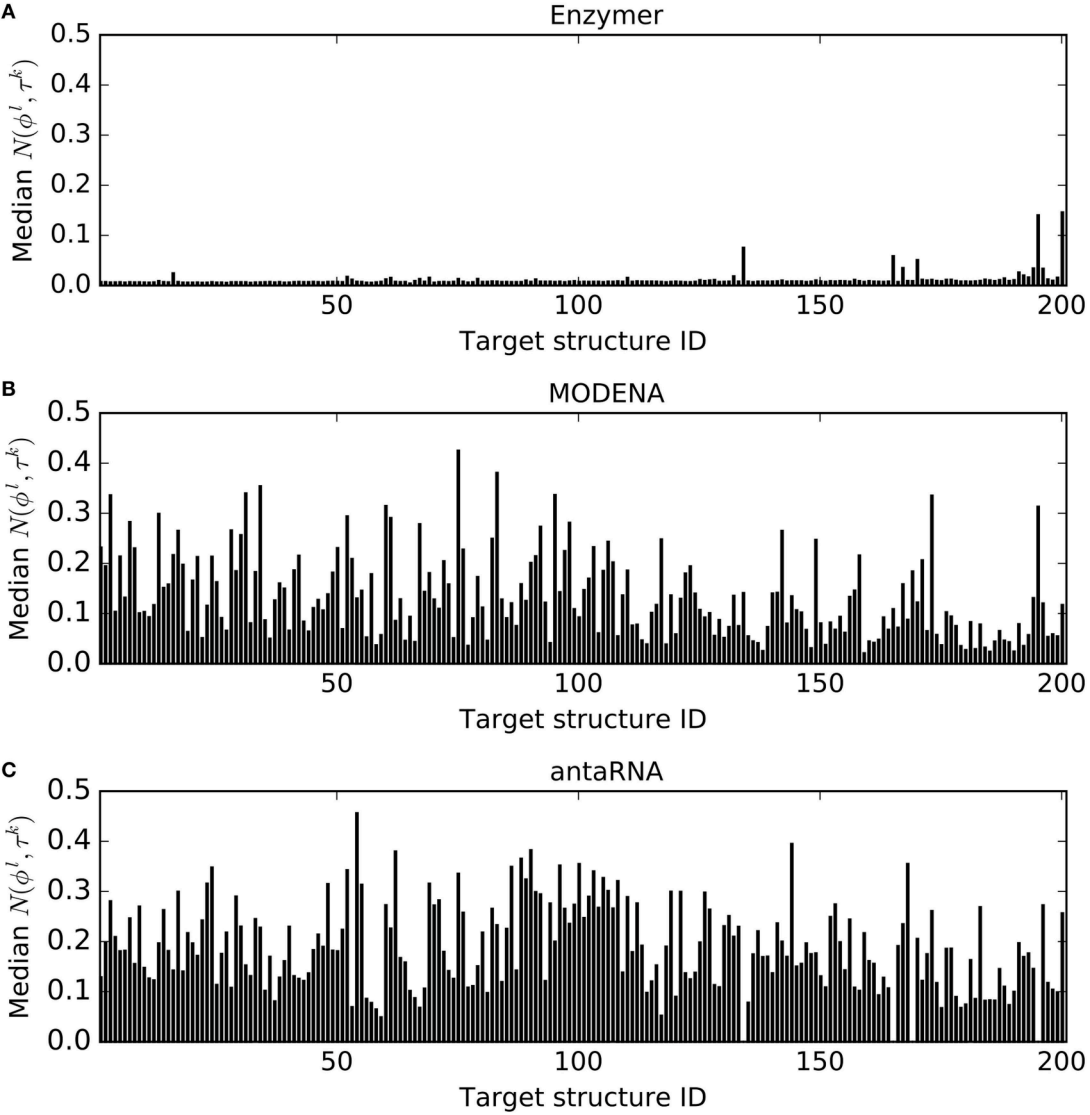
**Comparing normalized ensemble defect**. In each figure, each vertical bar represents the median *N*(ϕ^*l*^, τ^*k*^) obtained for each corresponding target. The results show Enzymer **(A)** outperformed both MODENA **(B)** and antaRNA **(C)** in 200 and 201 cases respectively. A binomial test statistic with 99% confidence suggests Enzymer delivers significantly better results compared to the other two methods. Furthermore, MODENA outperformed antaRNA in 155 cases and a binomial test static suggests that MODENA delivers significantly superior performance compared to antaRNA.

Figure 4 presents the median normalized ensemble defect values of the sequences generated by each method for each target structure. We observe Enzymer generated sequences with lower normalized ensemble defect and outperformed both MODENA and MODENA in 200 and 201 cases respectively. Furthermore, we also observe MODENA outperformed antaRNA in 155 cases. Respective binomial test statistics with *p*-values 1.25*e*^−58^ and 6.22*e*^−61^ suggest that Enzymer delivers superior performance compared to MODENA and antaRNA in generating sequences with lower ensemble defect. Furthermore, binomial test statistic with *p*-value 5.28*e*^−15^ suggests that MODENA delivers superior performance compared to antaRNA as well.

**Figure 5:**
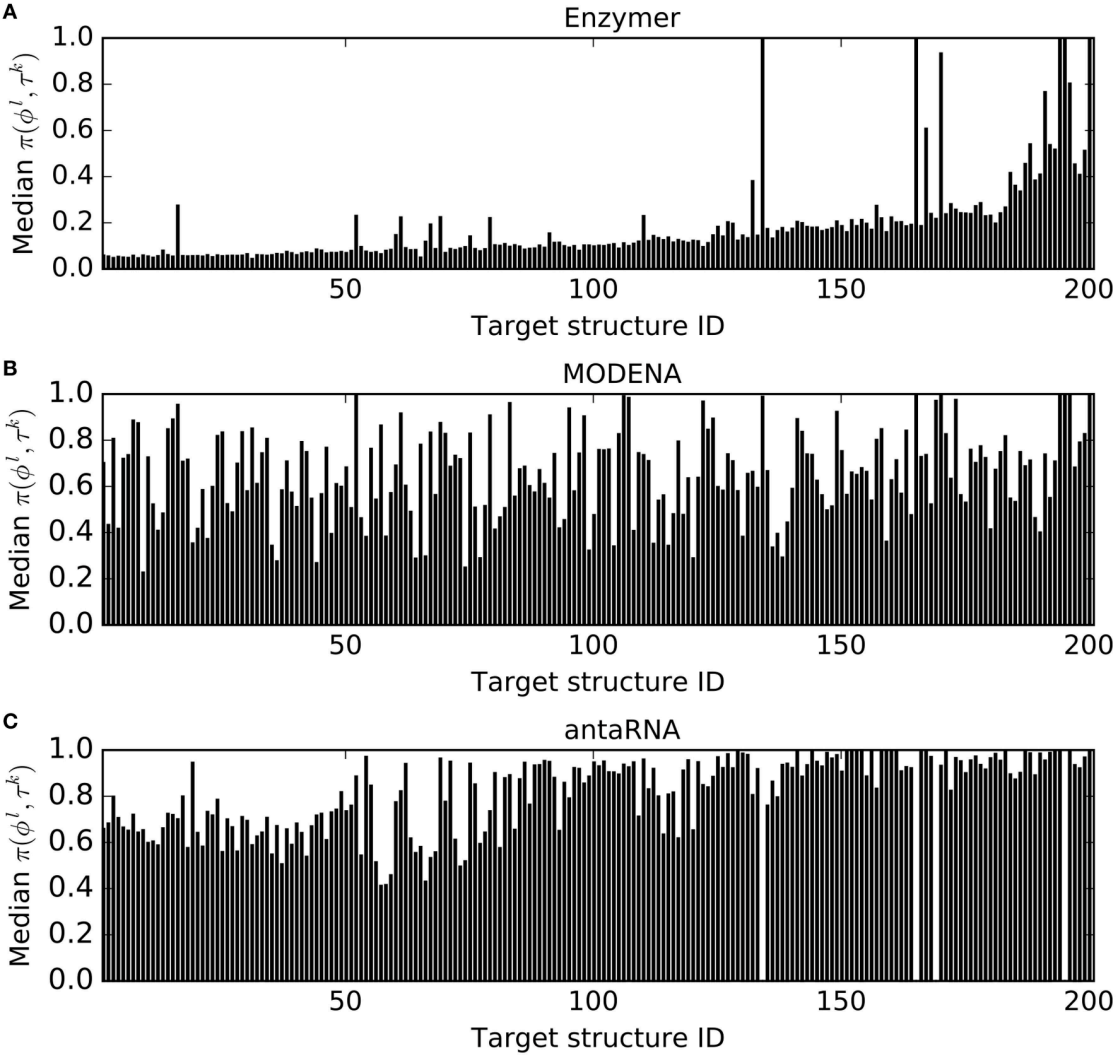
**Comparing probability defect values**. In each figure, each vertical bar represents the median π(ϕ^*l*^, τ^*k*^) obtained for each corresponding target. The results show Enzymer **(A)** outperformed both MODENA **(B)** and antaRNA **(C)** in 196 and 201 cases respectively. A binomial test statistic with 99% confidence suggests Enzymer delivers significantly better results compared to the other two methods. Furthermore, MODENA outperformed antaRNA in 153 cases and a binomial test static suggests that MODENA delivers significantly superior performance compared to antaRNA.

Figure 5 shows median probability defect values of the sequences generated by each method for each target structure. We observe Enzymer outperformed MODENA and antaRNA in 196 and 201 cases respectively. We also observe MODENA outperformed antaRNA in 153 cases. Respective binomial test statistics with *p*-values 1.66*e*^−51^ and 6.22*e*^−61^ suggest that Enzymer delivers superior performance compared to MODENA and antaRNA in generating sequences with lower probability defect. Furthermore, binomial test statistic with *p*-value 5.72*e*^−14^ suggests that MODENA delivers superior performance when compared to antaRNA as well.

**Figure 6:**
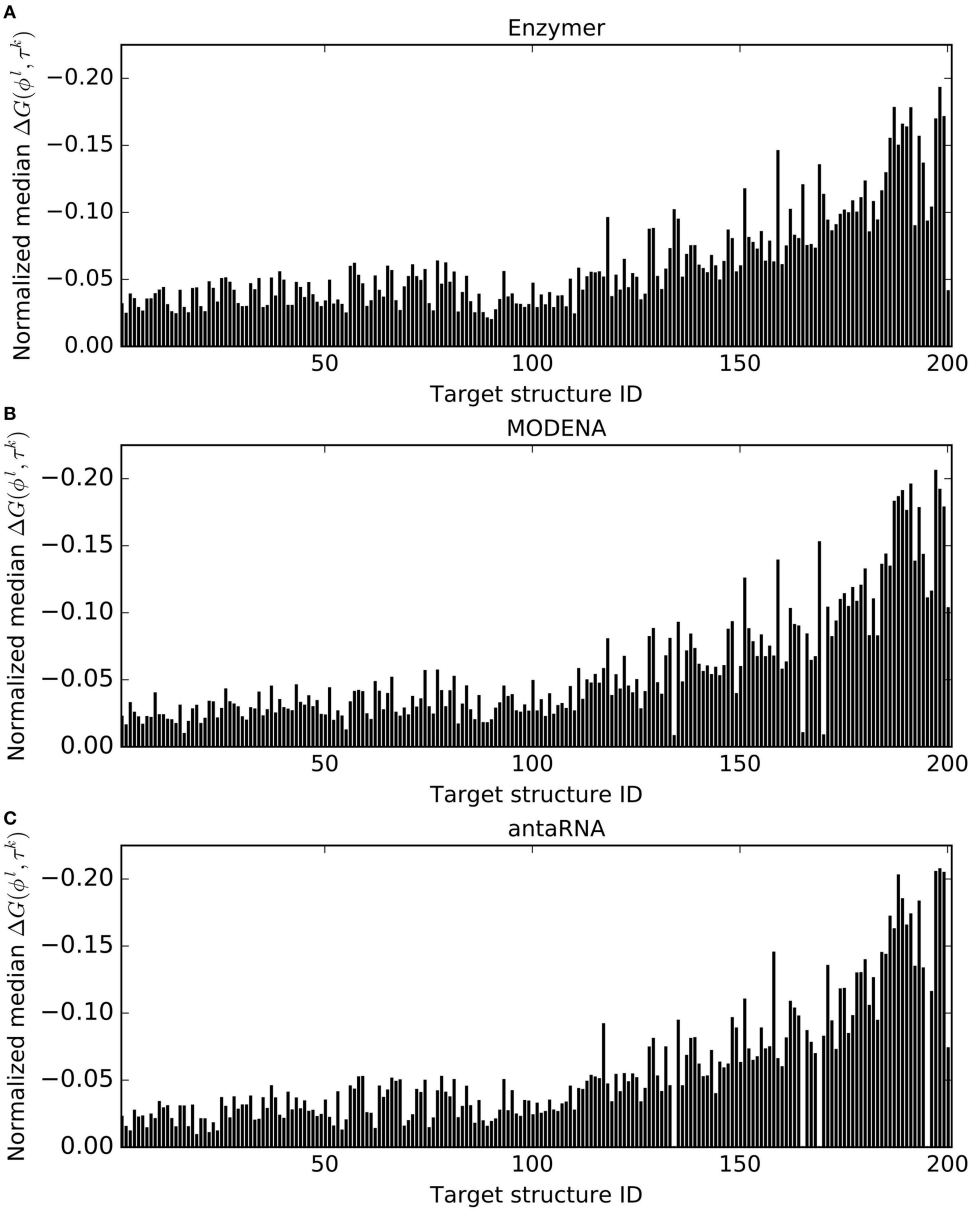
**Comparing normalized median free energy**. In each figure, each vertical bar represents the median Δ*G*(ϕ^*l*^, τ^*k*^) obtained for each corresponding target. The results show Enzymer **(A)** outperformed both MODENA **(B)** and antaRNA **(C)** in generating sequences with lower free energy in 102 and 198 cases respectively. A binomial test statistic with 99% confidence suggests Enzymer delivers significantly better results to antaRNA, however similar performance to MODENA. Furthermore, MODENA outperformed antaRNA in 195 cases and a binomial test static suggests that MODENA delivers significantly superior performance compared to antaRNA.

Figure 6 presents the normalized median free energy values of the sequences generated by each method. We observed Enzymer designed sequences with lower free energy compared to MODENA and antaRNA in 102 and 198 cases respectively. We also observe when compared with antaRNA, MODENA generated sequences with lower free energy in 195 cases. Respective binomial test statistics with with *p*-value 0.88 suggest Enzymer and MODENA generate sequences with similar free energy. However, respective binomial test statistics with *p*-values 8.42*e*^−55^ and 5.45*e*^−50^ suggest that both Enzymer and MODENA deliver superior performance compared to antaRNA in generating sequences that have lower free energy and therefore are thermodynamically more stable.

**Figure 7:**
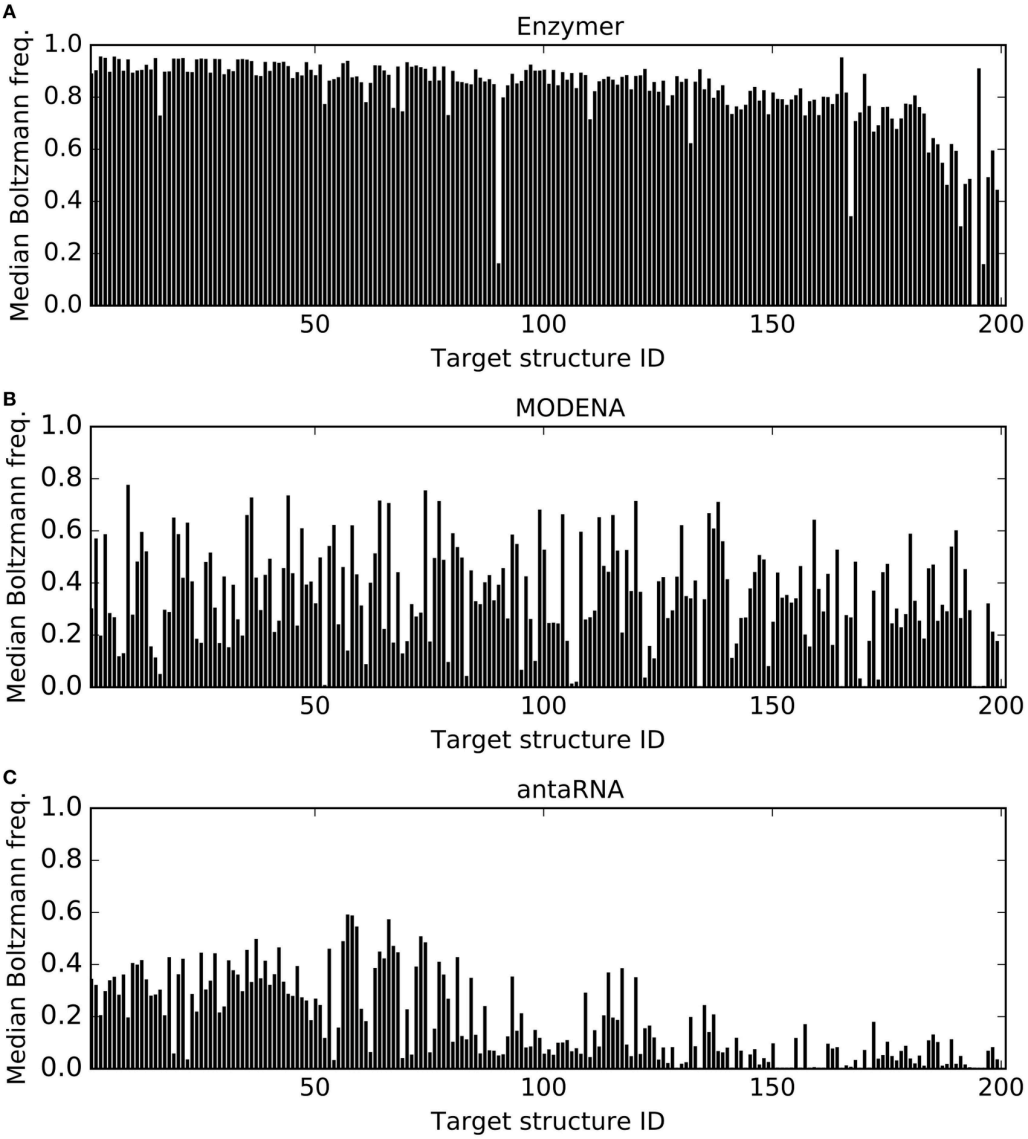
**Comparing median Boltzmann frequency**. In each figure, each vertical bar represents the median Boltzmann frequency obtained for each corresponding target. The results show Enzymer **(A)** outperformed both MODENA **(B)** and antaRNA **(C)** in 197 and 201 cases respectively. A binomial test statistic with 99% confidence suggests Enzymer delivers significantly better results compared to the other two methods. Furthermore, MODENA outperformed antaRNA in 153 cases and a binomial test static suggests that MODENA delivers significantly superior performance compared to antaRNA.

Figure 7 presents the median Boltzmann frequencies achieved by each of the methods. We observe Enzymer outperformed MODENA and antaRNA in generating sequences with higher Boltzmann frequency in 197 and 201 cases respectively. We also observe MODENA outperformed antaRNA in 153 cases. Respective binomial test statistics with *p*-values 4.19*e*^−53^ and 6.22*e*^−61^ suggest that Enzymer delivers superior performance compared to both MODENA and antaRNA in generating sequences that have higher Boltzmann frequency values. Moreover, binomial test statistic with *p*-value 5.72*e*^−14^ suggests that MODENA delivers superior performance compared to antaRNA.

**Figure 8:**
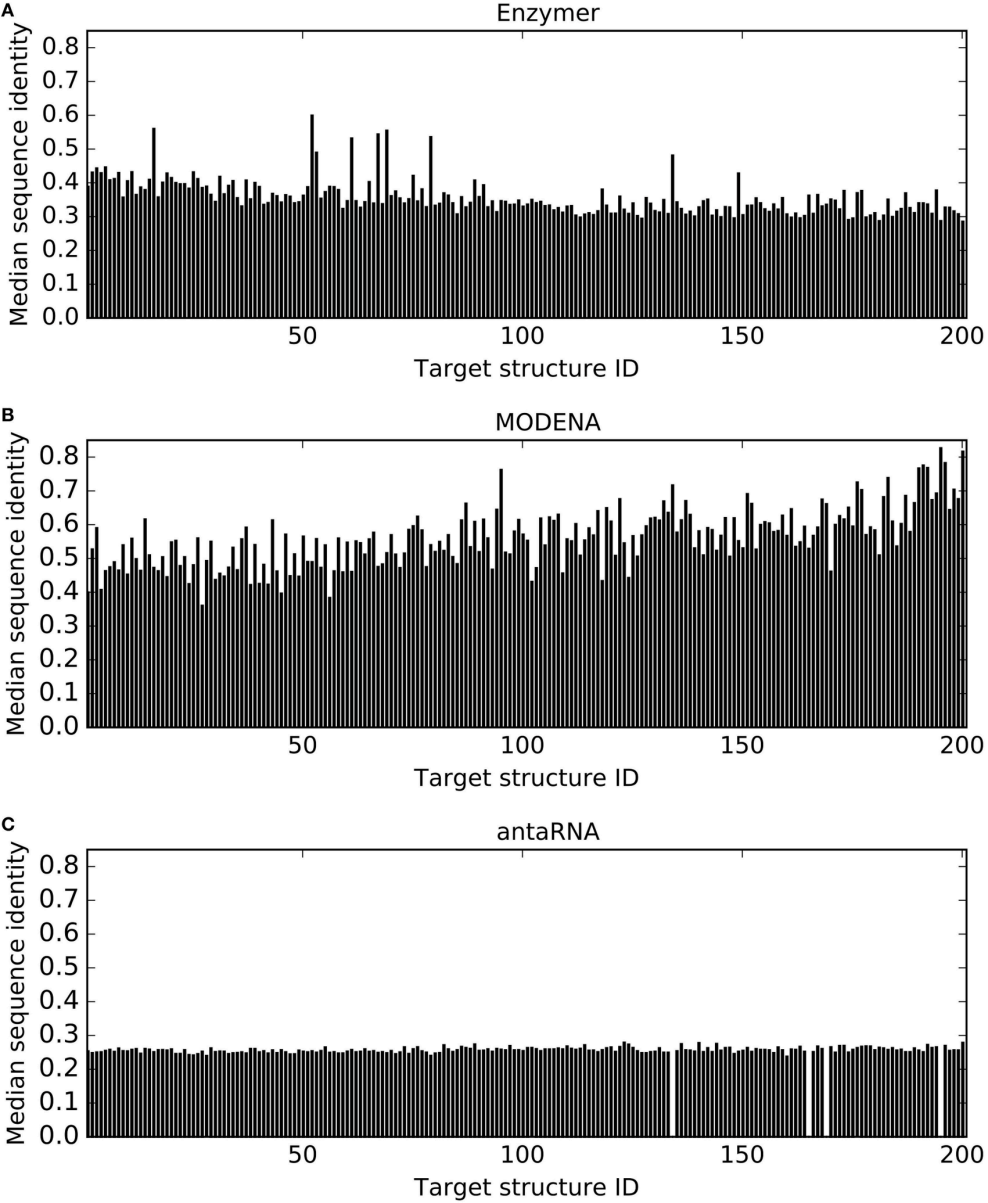
**Comparing sequence identity**. In each figure, each vertical bar represents the median sequence identity obtained for each corresponding target. For all 197 out of 201 cases where antaRNA **(C)** returned solutions, the median sequence identity was lower than Enzymer **(A)** as well as MODENA **(B)**. On the other hand in 193 cases Enzymer generated sequences with lower sequence identity when compared with MODENA. Binomial test statistic with 99% confidence suggests antaRNA outperforms the other methods in generating sequence populations that are more diverse while MODENA generates sequences with the lowest sequence diversity.

Figure 8 presents median sequence identity for sequence populations generated by each method. We observe antaRNA generated sequences with lower sequence identity in all 201 cases. When we compare Enzymer with MODENA, we observe Enzymer generated sequences with lower sequence identity in 193 cases. Binomial test statistics with *p*-value 6.22*e*^−61^ suggest antaRNA generates solution sets that have lower sequence identity than those sequences generated by Enzymer and MODENA. On the other hand binomial test with *p*-value 3.72*e*^−47^ suggests that MODENA generates solution sets with the lower degree of sequence diversity than the solution sets generated by Enzymer.

**Figure 9:**
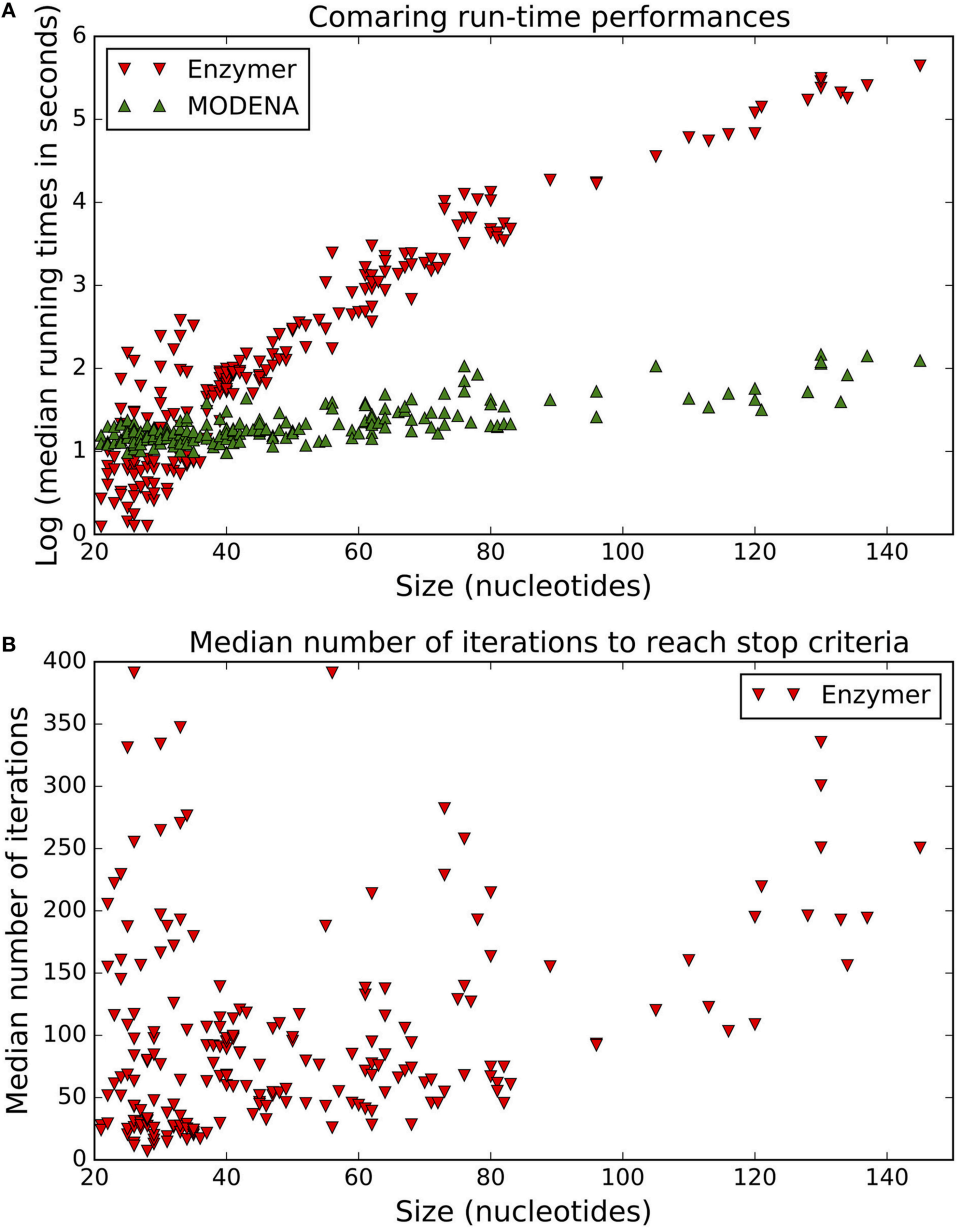
**Run-time performance of Enzymer**. **(A)** Comparing run-time performance of Enzymer and MODENA. **(B)** Enzymer reached the stop condition in less than 200 iterations for 179 out of 201 cases.

Figure 9A compares the run-time performance of Enzymer with MODENA. The y-axis quantifies the logarithm of the median running time required by each of the two methods to reach the corresponding stop criteria. The x-axis represents the size of the target structures in increasing order. As the size of the target structures grow, we observe a rapid rate of growth in the run-time requirement of Enzymer as opposed to a slower growth of run-time requirement for MODENA. The computationaly costly run-time requirement of Enzymer can be related to the expensive task of computing the partition function over the pseudoknotted ensemble in *O*(*n*^5^) time. We have omitted antaRNA from this figure because in our simulations we enforced antaRNA to run for the exact same amount of time it was required by Enzymer to reach the stop condition for each corresponding target structure. We note that the stop criteria for antaRNA is when the MFE defect becomes zero, however as Figure 3 shows there is no guarantee for antaRNA to reach the stop criteria and therefore an artificial cap on the maximum running time allowed must be applied. Figure 9B presents the median value for the number of iterations required for Enzymer to reach the stop criteria. We observe in 179 or 89% of the cases, the stop condition was reached in less than 200 iterations. Both MODENA and antaRNA have been omitted from Figure 9B. MODENA is omitted because it does not stop the optimization process unless it reaches the maximum number of iterations. We also omitted antaRNA because it was not possible to measure the total number iterations before antaRNA reached the stop condition.

**Figure 10:**
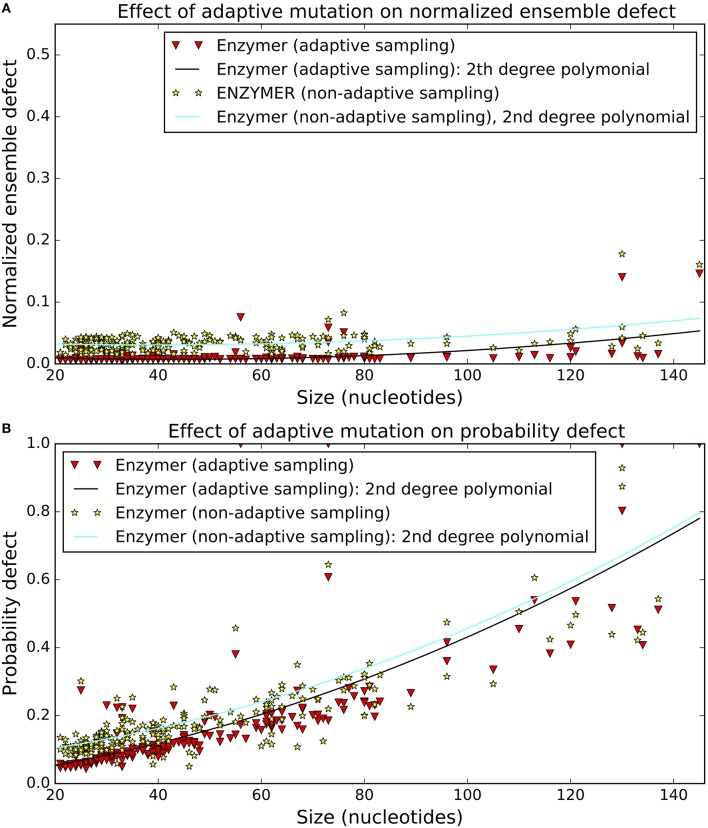
**Effect of adaptive sampling on defect**. The adaptive sampling strategy lowered the median normalized ensemble defect in 199 cases **(A)** and also lowered the median probability defect of the sequences in 181 cases **(B)**. Binomial test statistic with 99% confidence interval suggests for improving impact of the adaptive sampling strategy on both normalized ensemble defect and probability defect of the sequences we generated by Enzymer. For both figures the data was generated by setting *max*_*it* = 400.

The effect of the adding the adaptive sampling technique on normalized ensemble defect and probability defect values are presented in Figure 10. In order to make visual comparison possible, we also added the second degree curve to each dataset. We observe when we enabled the adaptive sampling schema (i.e., the third mutation operator) we reached lower normalized ensemble defect values in 199 out of 201 cases (Figure 10A). We also observe the adaptive sampling technique lowered the probability defect values in 181 out of 201 cases (Figure 10B). Respective binomial test statistics with *p*-values 1.26*e*^−56^ and 1.25*e*^−33^ strongly suggest that when the total number of iterations are kept constant (i.e., *max*_*it* = 400), the adaptive sampling strategy enables the algorithm to reach lower normalized ensemble defect and lower probability defect values and therefore improve on the run-time requirement of the algorithm.

### 3.3. Using naturally occurring motif sequences to design a hammerhead ribozyme

Hammerhead ribozymes are small self cleaving RNAs that promote strand scission by internal phosphodiaster transfer. In this section we describe a computational setup for the design of a *cis*-acting pseudoknotted Hammerhead ribozyme by using a set of naturally occurring and highly conserved nucleotides, which constitute a highly conserved Hammerhead motif. An RNA structural motif is defined as a collection of nucleotides that fold into a stable three dimensional (3D) structure, which can be found in naturally occurring RNAs in unexpected abundance.

**Figure 11:**
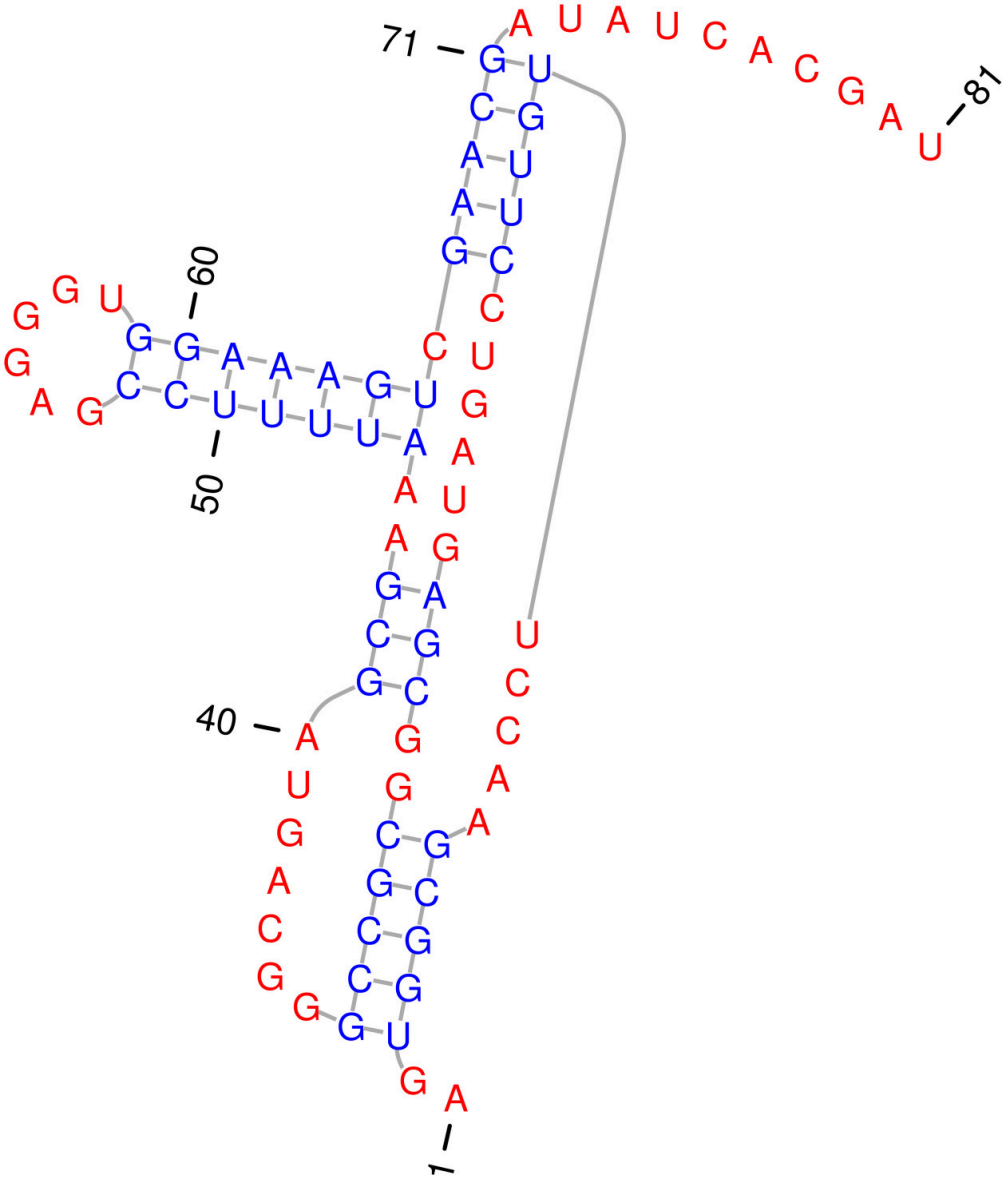
**Secondary structure of hammerhead ribozyme from mouse gut metagenome**. The stems are in blue and free nucleotides are in red. The 5 nucleotide long pseudoknot, starts at position 3 on stem 1. The shown sequence represents the *HHB*^1^ sequence designed by Enzymer. The secondary structure in standard dot bracket notation is presented by “..[[[[[…..(((((……(((..]]]]]…….)))..(((((((……))))))).)))))……….” and is extracted from Perreault et al. (2011). We generated this figure using PseudoViewer3 (Byun and Han, 2009).

Figure 11 shows the secondary structure of a Hammerhead ribozyme from mouse gut metagenome as reported by Perreault et al. (2011) and we will refer to it by *HH*. The reporting article also identifies the set of highly conserved motif nucleotides with ≥90% rate of conservation throughout the entire phylogenetic family of the ribozyme. Let the design template *t*_*HH*_ specify the highly conserved Hammerhead motif for the wild type *HH*. We adopt the motif specification from Perreault et al. (2011), and use it describe the RNA template sequence for *HH* by *t*_*HH*_ = *ooooooooooooooooCCUGAUGAGoooooooooooooooGCGAAAooooooooooooooooooUCGoooooooooooooo*. We used *t*_*HH*_ as the design template for Enzymer and use *HH* as the target structure and designed 8 sequences $\phi_{HH}^{l}$ where *l* = 1…8 for the Hammerhead ribozyme. We also set *max*_*it* = 400 and *f*_*stop*_ ≤ 0.01.

Table 1 presents the quality of the sequences we generated for *HH*. The last two rows show the mean and median values of the corresponding columns. Notably *f*_*stop*_ was satisfied in neither of the design trials however, the median normalized ensemble defect achieved was as low as 0.04. Interestingly, we observed that the median value for the free energy of the designed sequences is equal to 2.48*E* + 01 which is equivalent to the free energy of the wild type sequence of the Hammerhead ribozyme. The sequences we generated are presented in Table 1 of the Supplementary Materials section.

**Table 1:** **The data generated for the hammerhead ribozyme**.

**Annotation**	***N*($\phi_{HH}^{l}$, *HH*)**	**π($\phi_{HH}^{l}$, *HH*)**	**Δ*G*($\phi_{HH}^{l}$, *HH*)**	***max*_*it***
$\phi_{HH}^{1}$	4.01*E* − 02	5.41*E* − 01	−3.21*E* + 01	400
$\phi_{HH}^{2}$	4.97*E* − 02	6.33*E* − 01	−2.13*E* + 01	400
$\phi_{HH}^{3}$	5.02*E* − 02	6.66*E* − 01	−2.47*E* + 01	400
$\phi_{HH}^{4}$	4.34*E* − 02	5.85*E* − 01	−2.66*E* + 01	400
$\phi_{HH}^{5}$	4.43*E* − 02	5.76*E* − 01	−2.33*E* + 01	400
$\phi_{HH}^{6}$	4.99*E* − 02	6.44*E* − 01	−2.49*E* + 01	400
$\phi_{HH}^{7}$	4.29*E* − 02	5.73*E* − 01	−2.19*E* + 01	400
$\phi_{HH}^{8}$	5.38*E* − 02	7.05*E* − 01	−2.65*E* + 01	400
Mean	4.68*E* − 02	6.16*E* − 01	−2.52*E* + 01	400
Median	4.70*E* − 02	6.09*E* − 01	−2.48*E* + 01	400

## 4. Discussion

### 4.1. Summary of contributions

We presented Enzymer, a novel adaptive defect weighted sampling algorithm for the design of pseudoknotted RNA secondary structures. Enzymer (i) uses NUPACK to compute the equilibrium characteristics of RNA sequences, (ii) dynamically adapts the total number of positional mutations at each iteration during the run-time, and (iii) chooses target positions for mutation in respect to their type (free nucleotide, nested base pair or non-nested pair) as well as their positional contribution to ensemble defect of the sequence. To benchmark Enzymer, we used a biological dataset of naturally occurring pseudoknotted secondary structures from the PseudoBase library and compared our results with the state of the art MODENA and antaRNA.

### 4.2. Summary of results

Our benchmark dataset contains 201 naturally occurring pseudoknotted secondary structures of size 21–140 nucleotides. For each structure, we used Enzymer and generated 30 RNA sequences and compared our results with the results generated by MODENA and antaRNA. We showed that Enzymer efficiently explores the low ensemble defect mutational landscape of the candidate RNAs to design sequences that have lower ensemble defect, lower probability defect and higher Boltzmann frequency than those generated by MODENA and antaRNA. We also showed the sequences designed by our method have similar thermostability when compared to the sequences generated by MODENA but show better thermostability when compared the sequences generated by antaRNA. Furthermore, we showed our method succeeds more often than both MODENA and antaRNA do.

Furthermore, we observed that in 89% of the cases where the size of the target structure is bellow 140 nucleotides, our method can generate sequences with normalized ensemble defect value bellow 0.01 in less than 200 iterations. We also demonstrated that our adaptive sampling strategy causes the algorithm to reach the stop criteria in fewer number of iterations and therefore reduce the computational cost associated with the sampling process. Given our simulation results in respect to the run-time requirement of our approach, we conclude that our method is an excellent choice for the design of pseudoknotted RNA secondary structures of size up to 150 nucleotides. To our knowledge, there exists no other pseudoknotted RNA secondary structure designer algorithm that generates sequences that match the quality characteristics of sequences generated by Enzymer. Further experimentation will allow one to obtain a more accurate estimate about the applicability of Enzymer on larger and more diverse structures.

We emphasize that Enzymer extends the NUPACK design algorithm so that it include pseudoknots. However, if no pseudoknot is present in the target structure, our method will simply call the original NUPACK algorithm to generate sequences for pseudoknot-free targets.

### 4.3. Constrained sequence design to reengineer a hammerhead ribozyme

We used a naturally occurring Hammerhead motif and used Enzymer to reengineer a *cis*-acting Hammerhead ribozyme from the mouse gut metagenome. Our method achieved mean and median normalized ensemble defect values of 0.046 and 0.047 respectively. Future *in-vitro* experimentations will allow us to further analyze applicability of our algorithm as well as the applicability of the particular energy model we used to re-engineer functional *cis*-acting Hammerhead ribozymes.

### 4.4. Limitations

We note that the applicability of Enzymer is bound by the ability of NUPACK in recognizing different classes of pseudoknots. NUPACK realizes pseudoknots for single RNA strands such that the search space can be broken into all secondary structures that can be decomposed into two pseudoknot-free structures. Due to this limitation, when we used NUPACK to filter the original dataset, which was provided by Taneda (2012), the number of structures were reduced from 266 to 201. However, to our knowledge NUPACK is the only available computational framework, which can compute the partition function for a limited but biologically relevant class of pseudoknots. Hence, NUPACK is the best choice of the folding algorithm to design pseudoknotted RNAs with low ensemble defect, low probability defect and high thermostability.

### 4.5. Future work

To our knowledge neither Enzymer nor any other existing sequence designer algorithm exists, which can design RNA sequences for multi-strand and multi-target models such as the *trans*-acting glmS ribozyme described by Klein and Ferré-D'Amaré (2006) or the oligonucleotide-sensing allosteric ribozyme based logic gates such as the ones described by Penchovsky and Breaker (2005) if pseudoknots are present.

One can use NUPACK to compute the equilibrium characteristics of pseudoknot-free complexes of interacting RNA species (Wolfe and Pierce, 2014), or use NanoFolder (Bindewald et al., 2011) to predict base pairings of pseudoknotted complexes of interacting RNA species. As a future work, we intent to use NUPACK and NanoFolder as folding algorithms to build on our adaptive defect weighted sampling algorithm in order to include the ability to design RNA sequences for multi-strand and multi-target secondary structures where pseudoknots can be present in single stranded forms. Such improvement will open door to design oligonucleotide sensing genetic networks that implement more complex modular interactions such as networks of interacting RNA species where each single stranded RNA species can include pseudoknots.

## Author contributions

KZ: Developed the methodology and implemented the software, generated results, conducted the analysis and wrote the manuscript in its entire form. KZ also revised the manuscript to address the issues raised by the reviewers. GB: Provided oversight to the research process, provided comments and corrective remarks regarding the methodology and the analysis. NK: Provided supervision for research process related to this article, monitored the discussion sessions, read and provided corrective remarks about the methodology, implementation and analysis.

### Conflict of interest statement

The authors declare that the research was conducted in the absence of any commercial or financial relationships that could be construed as a potential conflict of interest.
