# Supplementary material for: An Adaptive Defect Weighted Sampling Algorithm to Design Pseudoknotted RNA Secondary Structures
Source: Front Genet. 2016 Jul 22;7:129. doi: 10.3389/fgene.2016.00129 (PMC4956659; doi:10.3389/fgene.2016.00129)
Supplement: Supplementary file 1 [file DataSheet1.PDF]

Supplementary Material: An adaptive defect weighted sampling algorithm to design pseudoknotted RNA secondary structures

Kasra Zandi*, Gregory Butler and Nawwaf Kharma*

*Correspondence:
Kasra Zandi, Nawwaf Kharma
k_zandi@encs.concordia.ca, kharma@ece.concordia.ca

1 SUPPLEMENTARY DATA

Figure 1 presents the size distribution of the secondary structures from our benchmark data set.

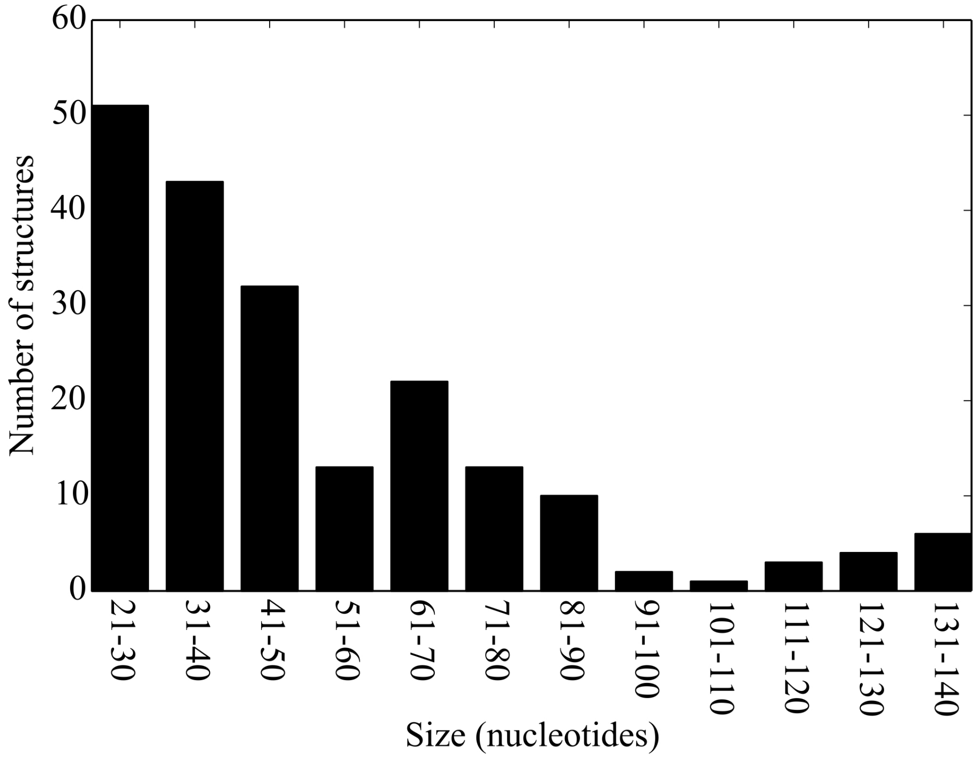

Supplementary Figure 1. Size distribution of the secondary structures in the benchmark dataset.

Table 1 presents the sequences we designed using `Enzymer` for the mouse gut metagenome Hammerhead ribozyme.

Supplementary Table 1. The conserved nucleotides are in red.

Annotation	Designed RNA sequence
ϕ^{1}_{HH}	CCGUCGCAAA AAGGGUCCUG AUGAGCAAGC GACAAAAAAA GCGAAACACC GCGAAAAAGC GGUGUCGACC CGAGAAAAAA G
ϕ^{2}_{HH}	CUGAUAGACC CCGGAUCCUG AUGAGCUACU AUCCCUAAAU GCGAAACACA GGCAUGAACC UGUGUCGAUC CUAUAAAACC C
ϕ^{3}_{HH}	CCCCUCUAAA AAGGGACCUG AUGAGCCCAG AGGAAAAAAC GCGAAAGGCU GCUAAAGUGU AGUCUCGUCC CAAACAACAU A
ϕ^{4}_{HH}	CAGUUCGAAA AAGCCUCCUG AUGAGCAACG AACAAACCUA GCGAAACCGU GGUUAACUCC AUGGUCGAGG CGACAAAAAA U
ϕ^{5}_{HH}	AACGGAGCCC UUCCGCCCUG AUGAGCAACU CUGAAUAAAA GCGAAACUGU AGAACUACCU ACGGUCGGCG GUUUUCUAUA C
ϕ^{6}_{HH}	CCCCUCGAAA AAGUGUCCUG AUGAGCAACG AGGAACCCCC GCGAAAGGCG UGAAAUACCG CGUCUCGACA CAAGAGAAAA G
ϕ^{7}_{HH}	ACGGACCACC CCCAGUCCUG AUGAGCAAGG UCCAAAAAAA GCGAAACUUG AUGUAAUAGU UAAGUCGACU GAAAAAAACC A
ϕ^{8}_{HH}	ACGGAGGGUG UGGGGCCCUG AUGAGCUGCC UCCUUGAAUU GCGAAAGUUG GAAUGAUCUC UAACUCGGCC CGUUGAGUUG U
ϕ^{wild}_{HH}	GGUACCGAAU AAAUCCCCUG AUGAGCAACG GUGAGAGCCG GCGAAACUAC CCAAACAAGG GUAGUCGGGA UAGUACCAUA A
Design template t_{HH}	oooooooooo ooooooCCUG AUGAGooooo oooooooooo GCGAAAoooo oooooooooo ooooUCGooo oooooooooo o
Secondary structure	..[[[[[.....(((((......(((..]]]]].......)))..(((((((......))))))).)))))..........
